# Fluid restriction vs liberal intake in patients with heart failure: a meta-analysis of randomized trials

**DOI:** 10.1093/eschf/xvaf004

**Published:** 2026-01-08

**Authors:** Agata Bielecka-Dabrowa, Maciej Banach, Danisha Kumar, Vikash Jaiswal

**Affiliations:** Department of Cardiology and Congenital Diseases of Adults, Polish Mother's Memorial Hospital Research Institute (PMMHRI), Lodz, Poland; Department of Preventive Cardiology and Lipidology, Medical University of Lodz, Lodz, Poland; Department of Cardiology and Congenital Diseases of Adults, Polish Mother's Memorial Hospital Research Institute (PMMHRI), Lodz, Poland; Department of Preventive Cardiology and Lipidology, Medical University of Lodz, Lodz, Poland; Ciccarone Center for the Prevention of Cardiovascular Disease, Johns Hopkins University School of Medicine, Baltimore, MD, USA; Department of Internal Medicine, Dow University of Health Sciences, Karachi, Pakistan; Department of Medicine, AMA School of Medicine, Makati, Philippines

**Keywords:** Heart failure, Fluid restriction, Euvolemia

## Abstract

**Introduction:**

In the absence of enough supportive evidence, the US and European guidelines for the diagnosis and treatment of acute and chronic HF provide only general recommendations supporting fluid restriction (FR) for selected patients with symptomatic HF. We aimed to evaluate the risk of all-cause mortality, hospital readmissions and change in BNP, sodium and perceived thirst in HF patients with FR vs liberal fluid intake.

**Methods:**

We performed a systematic literature search on PubMed, Embase, and Clinicaltrials.gov for relevant randomized controlled trials (RCTs) from inception until June, 2025. Risk ratios (RR), weighted mean differences (WMD) and 95% confidence intervals (CI) were pooled using a random-effects model with the Hartung–Knapp–Sidik–Jonkman (HKSJ) adjustment, and between-study variance (τ²) was estimated with restricted maximum likelihood (REML).

**Results:**

A total of 9 RCTs with 1271 patients (614 in the FR group and 657 in the non-FR group; mean follow-up: 196.33 days) were included in the study. The mean age of patients among FR and non-FR groups was 69.48 and 68.67 years. Pooled analysis showed a statistically significant reduction in the risk of all-cause mortality with restricted fluid intake compared with liberal intake (RR = 0.54; 95% CI: 0.31–0.94; *P* = 0.03). There were no significant difference between restricted and liberal fluid intake groups (RR = 0.67; 95% CI: 0.28 to 1.65; *P* = 0.31) regarding heart failure hospital readmissions. No significant differences were observed in perceived thirst (WMD = −6.89; 95% CI: −22.86 to 9.08; *P* = 0.26), serum BNP levels (WMD = 54.09 pg/mL; 95% CI: −316.86 to 425.04; *P* = 0.71), or sodium levels (WMD = 1.42 mmol/L; 95% CI: −0.68 to 3.51; *P* = 0.15).

**Conclusion:**

Fluid restriction reduces the risk of all-cause mortality but not HF rehospitalizations in HF patients. Further studies are warranted to definitively confirm the present findings and result on the suitable changes in recommendations and clinical practice.

## Graphical Abstract

**Graphical Abstract:**
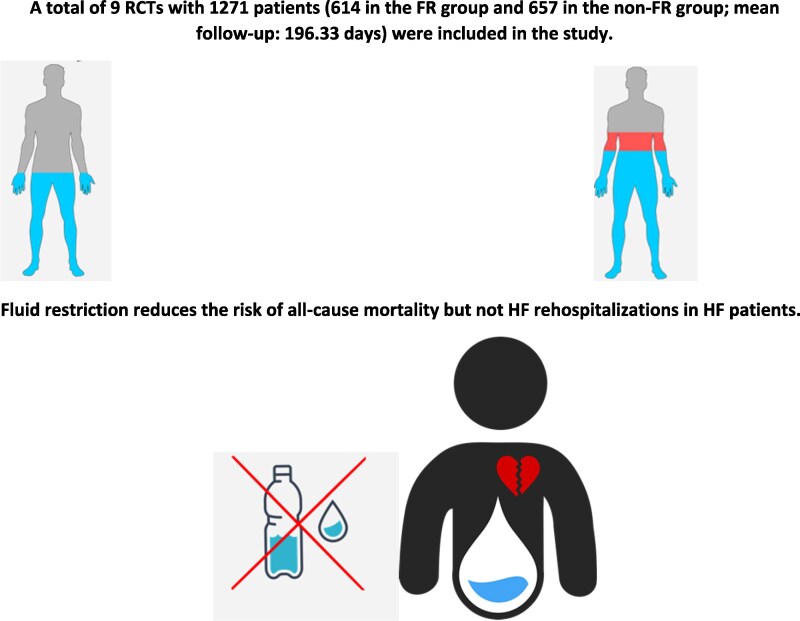
Summary of the meta-analysis evaluating the risk of all-cause mortality, hospital readmissions and change in BNP, sodium, and perceived thirst in heart failure patients with restricted fluid intake vs liberal fluid intake. Pooled analysis of 9 RCTs with 1271 patients showed a statistically significant reduction in the risk of all-cause mortality with restricted fluid intake compared to liberal intake but no significant differences in heart failure hospital readmissions.

## Introduction

Heart failure (HF) is a leading cause of morbidity and mortality, and causes high health-care-related costs, posing a great burden on both patients and society.^1^

In the treatment of HF, nonpharmacological treatments, especially self-care, are considered important and strongly recommended in guidelines in Europe despite optimal pharmacological treatment.^2^ In general, a normal fluid intake falls within the range of 1.5 to 2.5 L/day, corresponding to 15–30 mL/kg/day. A more liberal fluid policy is considered to involve an intake of more than 2.5–3.0 L/day, whereas a restrictive fluid policy typically entails an intake of < 1–1.5 L/day.^3^ Fluid restriction was traditionally considered a cornerstone of nonpharmacological management in HF patients. Some patients with HF are still advised to limit fluid intake in clinical practice.^3^ The 2021 ESC HF guidelines recommend avoiding large volumes of fluid intake for all patients with HF with fluid restriction of 1.5–2 L/day which may be considered in patients with severe HF/hyponatremia to relieve symptoms and congestion’ (although it is a general recommendation, no level of evidence is provided).^2^ These recommendations derive from the pathophysiological changes in the sympathetic nervous system, the renin–angiotensin–aldosterone system (RAAS), the vasopressin axis, and vasodilatory/natriuretic pathways in patients with HF.^4^ Although fluid overload is inextricably linked to heart failure (HF), dehydration is also possible in this population. It remains unknown whether the evidence supports fluid restriction in patients with HF. This recommendation has largely been based on expert opinion. Very limited data with low level of evidence suggests that fluid restriction may reduce the risk for HF hospitalization, whereas other reports suggest it is not helpful for improving outcomes and is worse for patients’ quality of life.^4^

In the position paper on self-care of HF patients [published by Heart Failure Association of the European Society of Cardiology (ESC)] ‘optimal fluid and salt management in patients with HF’ was listed as a recommendation for future research.^3^ In the 2021 ESC Guidelines for the diagnosis and treatment of acute and chronic HF ‘evidence on the effects of fluid restriction’ was listed as a gap in evidence.^2^ Similar statement can be found in the 2022 American Heart Association (AHA) Guideline for the Management of Heart Failure.^5^ Thus, we aimed to evaluate the risk of all-cause mortality, hospital readmissions and change in BNP, sodium, and perceived thirst in HF patients with FR vs liberal fluid intake.

## Methods

This meta-analysis was conducted and reported following the PRISMA (Preferred reporting items for systematic review and Meta-analysis) 2020 guidelines.^6^ A comprehensive systematic literature search was conducted in PubMed, Embase, and Clinicaltrials.gov utilizing predefined MESH terms by using ‘AND’ and ‘OR’. The following search terms were used: [‘Heart Failure’(Mesh) OR ‘heart failure’(tiab) OR ‘cardiac failure’(tiab) OR ‘congestive heart failure’(tiab) OR CHF(tiab)] AND [‘Fluid Therapy’(Mesh) OR ‘Water-Electrolyte Balance’(Mesh) OR ‘fluid restriction’(tiab) OR ‘restricted fluid’(tiab) OR ‘fluid intake’(tiab) OR ‘liberal fluid’(tiab) OR ‘liberal intake’(tiab) OR ‘fluid management’(tiab) OR ‘water restriction’(tiab)] AND [‘Randomized Controlled Trial’(Publication Type) OR ‘Randomized Controlled Trials as Topic’(Mesh) OR randomized(tiab) OR randomized(tiab) OR trial(tiab) OR RCT(tiab)]. Our meta-analysis aimed to include all the available randomized controlled trials published till June 2025, which compares patients with restricted fluid intake with control groups of patients with liberal fluid intake. Studies reporting outcomes of interest, RCTs, and Adults (≥18 years) diagnosed with heart failure were sought to be included in the analysis. Studies on animals, patients <18 years, conference abstracts, reviews, and single-arm studies were excluded from the analysis.

### Risk of bias assessment

To assess the quality of each included study, we used Cochrane RoB 2 (risk-of-bias tool for randomized trials and cross-over trials) tool. The RoB 2 tool facilitates risk of bias assessment of studies that use randomization as a method for selecting participants, and rates studies as ‘low concerns,’ ‘some concerns,’ or ‘high concerns,’ based on set criteria (Supplementary Figs. S1 and S2).^7^ Domains recorded included: randomization process, deviations from the intended outcomes or bias arising from period and carryover effects as in the case of cross-over trial, missing outcome data, measurement of the outcome and selection of the reported result. Risk of bias assessment was performed by two authors independently. To rate the certainty of evidence for each outcome, we used the GRADE (Grading of Recommendations, Assessment, Development and Evaluations) approach. Two authors independently performed GRADE assessment. Any disparity was solved by mutual consensus and in consultation with other authors.^8^

### Statistical analysis

Regarding the binary variables, the effect size was presented as risk ratio (RR) with a 95% confidence interval (CI), using a random-effects model. For the other outcome measures, such as continuous variables, we adopted the weighted mean difference (WMD) for estimating the effect size, using a random-effects model once again. Given that fewer than 10 studies were included in the analysis, we applied the Hartung-Knapp-Sidik-Jonkman (HKSJ)^9,10^ method to obtain more accurate and conservative estimates of the pooled effect and its confidence intervals. This method is recommended in meta-analyses with small numbers of studies, as it better accounts for uncertainty in the between-study variance estimate. Heterogeneity was assessed using the I² statistic, τ² [estimated via restricted maximum-likelihood (REML)], and Cochran’s Q test to represent the percentage of variability due to between study variability. Values for *I*^2^ of <25%, 25%–50%, and more than 50% were rated as low, moderate, and high amounts of heterogeneity, respectively. Results were regarded as statistically significant if the *P*-value was < 0.05.^11^ Furthermore, to identify potential outliers, we conducted a ‘leave-one-out’ sensitivity analysis, recalculating pooled effect sizes and heterogeneity results after sequentially omitting one study at a time.^12^ Due to the small number of included studies (<10) for each outcome, formal assessment of publication bias using funnel plots or statistical tests (e.g. Egger’s test) was not performed, as such methods are considered unreliable with small sample sizes and may yield misleading results. All the analyses were conducted by using Review Manager Web version 9.10.0: 03 July 2025 (Cochrane Collaboration, Copenhagen, Denmark).^13^

## Results

**Figure 1:**
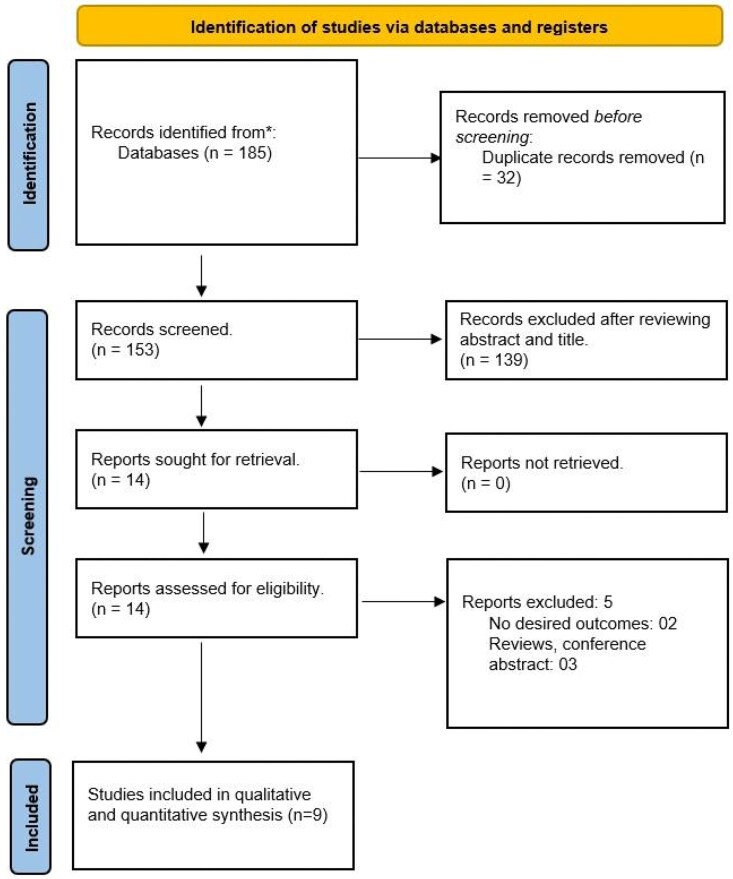
PRISMA flow diagram of search among different databases.

The initial search strategy yielded 185 articles of which 32 duplicates were removed, and 139 articles were excluded after the title and abstract screening. Finally, 14 articles were sought for retrieval, and 5 studies were excluded due to the lack of outcomes or a conference abstract. A total of 9 RCTs with 1271 patients (614 in the FR group and 657 in the non-FR group) were included in the study (*Figure 1*).^14–22^ The mean age of patients among FR and non-FR groups was 69.48 and 68.67 years (*Table 1*). The mean follow-up duration was 196.33 days.

**Table 1:** Summary of selected articles

Author	Population	Country	Defining parameters	Follow-up	Sample size	Overall risk of bias
**Travers 2007^14^**	18 years of age or older with a confirmed diagnosis of class IV HF	Ireland	Liberal fluid intake (free according to thirst) vs. fluid restriction (1litre daily)	2 weeks	67	High risk- participants were withdrawn for worsening renal function. More patients withdrawn from FR group.
**Holst 2008^15^**	CHF with left ventricular ejection fraction (LVEF) ≤45% and stable condition without clinical signs of significant fluid overload	Sweden	Liberal fluid intake (30–35 mL/kg body weight/day) vs. fluid restriction (1.5 L daily)	16 weeks	130	Some concerns-While most domains are low risk, the absence of a washout period and lack of adherence-adjusted analysis introduce moderate concerns.
**Paterna 2009^16^**	Compensated HF who were hospitalized previously (within 30 days) for recently decompensated HF	Italy	Liberal fluid intake (2 L per day) vs. fluid restriction (1 L daily) plus restriction	24 weeks	104	Low risk-All domains were low, except for selection of reported results due to no pre-specified analysis plan.
**Aliti 2013^17^**	ADHF and systolic dysfunction (LVEF) ≤45%	Brazil	Liberal fluid intake (at least 2.5 L per day) vs. fluid restriction (0.8 L daily) plus sodium restriction	4 weeks	75	Low risk-All domains were low, except for selection of reported results due to no pre-specified analysis plan.
**Albert 2013^18^**	Acute decompensation of chronic HF were recruited if they had hypervolemic or euvolemic hyponatremia, defined as a serum sodium <137 mmol/dL any time during the hospitalization episode	USA	Liberal fluid intake (no specific fluid restrictions) vs. fluid restriction (1 L daily)	8 weeks	46	High risk-Due to substantial deviations from intended interventions, self-reported QOL outcome, and absence of a pre-specified analysis plan.
**Philipson 2013^19^**	CHF, documented LV dysfunction (EF ≤40%, or >40% and a history of hospitalization for HF	Sweden	Liberal fluid intake (30–35 mL/kg body weight/day) vs. fluid restriction (1.5 L daily) plus sodium restriction	12 weeks	97	High risk-Due to unblinded subjective outcomes and absence of a pre-specified analysis plan
**Machado 2018^20^**	Adult patients with a diagnosis of HF and left ventricular ejection fraction (EF) ≥50% admitted to hospital for decompensation of HF	Brazil	Sodium (2 g salt)/day and fluid restriction 0.8 L daily vs unlimited fluid intake and about (10 g salt)/day	4 weeks	53	Low risk-All domains were low, except for selection of reported results due to no pre-specified analysis plan
**Colin Ramirez 2010^21^**	Adult patients with HF based on reduced systolic and diastolic function in echocardiography	Canada	Sodium-restriction and fluid-restriction (<1500 mL/d) vs no special recommendations	48 weeks	195	High risk- Due to non-ITT analysis, substantial exclusions, and selective reporting without protocol transparency
**Herrmann 2025^22^**	Chronic heart failure, Age ≥18 years, treated for chronic HF for >6 months prior to randomization. NYHA class II or III HF symptoms	Netherland	liberal fluid intake (no specific fluid restrictions, allowing to drink according to their thirst) or fluid restriction up to 1500 mL per day	6 months	504	High risk-Due to unblinded participants and subjective outcome measurement, both likely to favor the liberal fluid intake group.

ADHF, acute decompensated heart failure; CHF, congestive heart failure; EF, ejection fraction; FR, Fluid restriction; HF, heart failure.

### Risk of bias assessment

Of the nine included trials, three were judged at low risk of bias (Paterna 2009, Aliti 2013, Machado 2018), one study raised some concerns (Holst 2008), and five were considered at high risk of bias (Travers 2007, Albert 2013, Philipson 2013, Colin Ramirez 2010, Herrmann 2025). Most studies adequately addressed the randomization process and had low risk for missing outcome data; however, common concerns arose from deviations from intended interventions, lack of blinding with subjective outcomes, and selective reporting without prespecified protocols. High risk was particularly evident in studies with substantial participant withdrawal (Travers 2007), reliance on self-reported outcomes (Albert 2013), and absence of intention-to-treat analysis (Colin Ramirez 2010). Furthermore, unblinded participants combined with subjective outcome measurement posed a risk in Philipson 2013 and Herrmann 2025. Holst 2008 was rated as having some concerns due to the absence of a washout period and lack of adherence-adjusted analysis, despite otherwise low-risk domains (Supplementary Figs. S1 and S2). The Grading of Recommendations Assessment, Development and Evaluation (GRADE) tool revealed the overall strength of evidence for all 5 outcomes to be moderate (Supplementary Table S1).

### All-cause mortality

**Figure 2:**
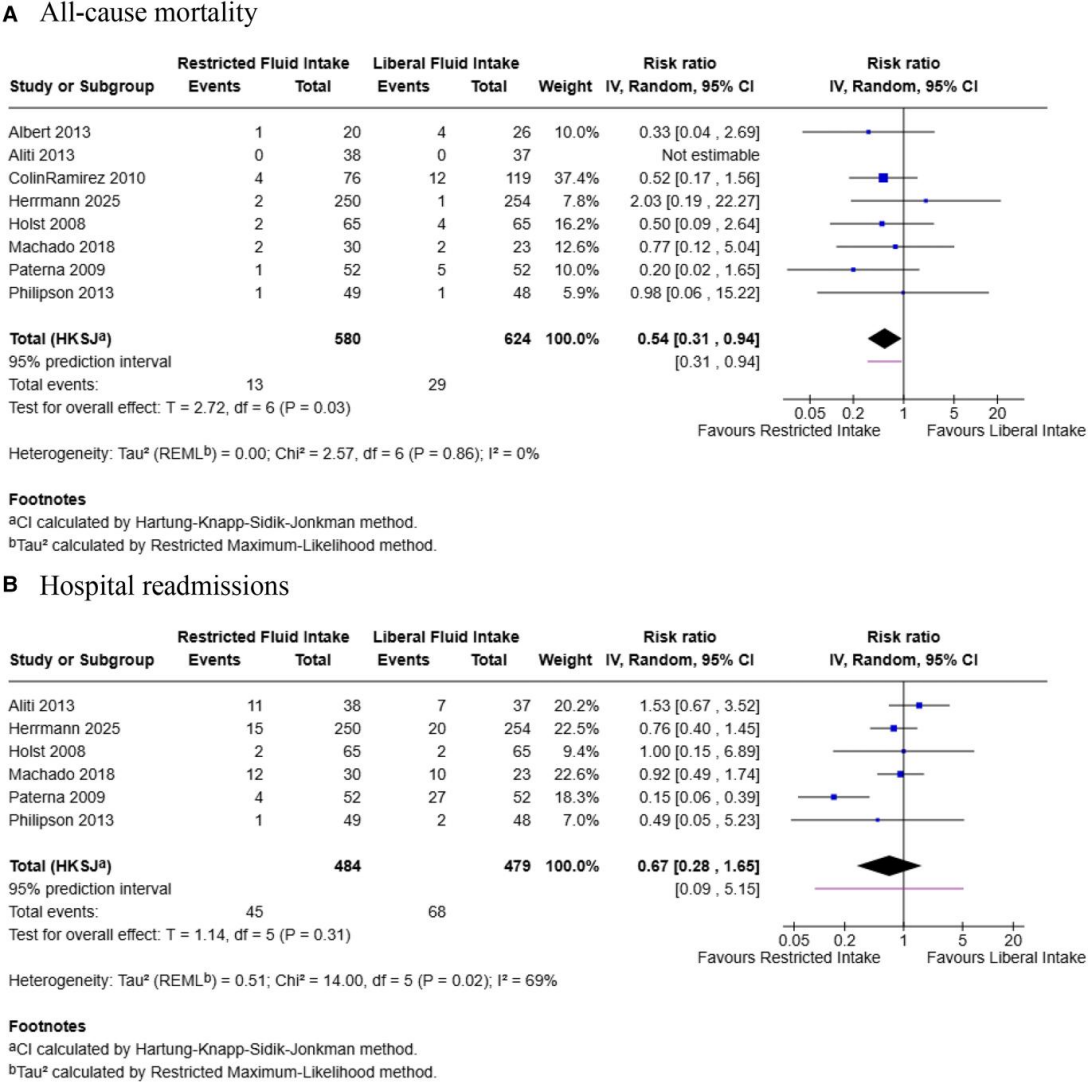
Forest plot summarizing results of (*A*). All-cause mortality, and (*B*) Hospital readmissions

Pooled estimates of eight out of nine studies showed that restricted fluid intake was associated with a significantly lower risk of all-cause mortality compared with liberal fluid intake [RR: 0.54 (95% CI: 0.31–0.94); *P* = 0.03; *I*² = 0%] (*Figure 2*, Panel *A*). Between-study heterogeneity was negligible (τau² = 0.00), and no single study disproportionately influenced the overall result in sensitivity analyses, indicating consistency of effect across trials.

### Hospital readmissions

Six out of nine trials reported data on hospital readmissions. Pooled analysis showed no significant difference between restricted and liberal fluid intake groups [RR = 0.67 (95% CI: 0.28 to 1.65); *P* = 0.31] (*Figure 2*, Panel *B*). The pooled effect slightly favored restricted fluid intake, but the difference was not statistically significant. There was moderate heterogeneity across studies (*I*² = 69%, τau² = 0.51). Leave-one-out sensitivity analysis identified the trial by Paterna et al. (2009) as a major source of heterogeneity. After excluding this study, the pooled risk ratio remained nonsignificant [RR = 0.92 (95% CI: 0.54 to 1.56); *P* = 0.75], and heterogeneity was substantially reduced (*I*² = 0%, τau² = 0.00) (Supplementary Fig. S3).

### Perceived thirst

**Figure 3:**
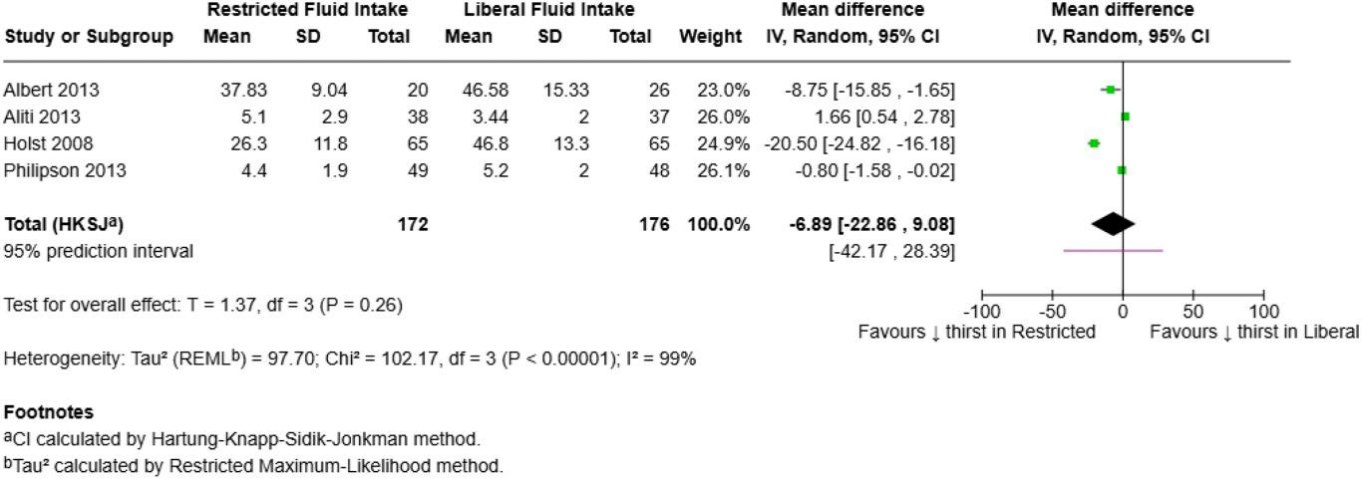
Forest plot summarizing results of perceived thirst at follow-up.

Four out of nine studies reported on perceived thirst at follow-up. Pooled analysis showed no significant difference between restricted and liberal fluid intake groups in terms of perceived thirst at follow-up [WMD = −6.89, (95% CI: −22.86 to 9.08); *P* = 0.26] (*Figure 3*). Substantial heterogeneity was observed across studies (*I*² = 99%, τau² = 97.70), indicating considerable variability in effect estimates. Sensitivity analysis did not meaningfully reduce heterogeneity, and the direction of the pooled effect remained consistent, favoring restricted fluid intake.

### Mean Serum BNP levels

**Figure 4:**
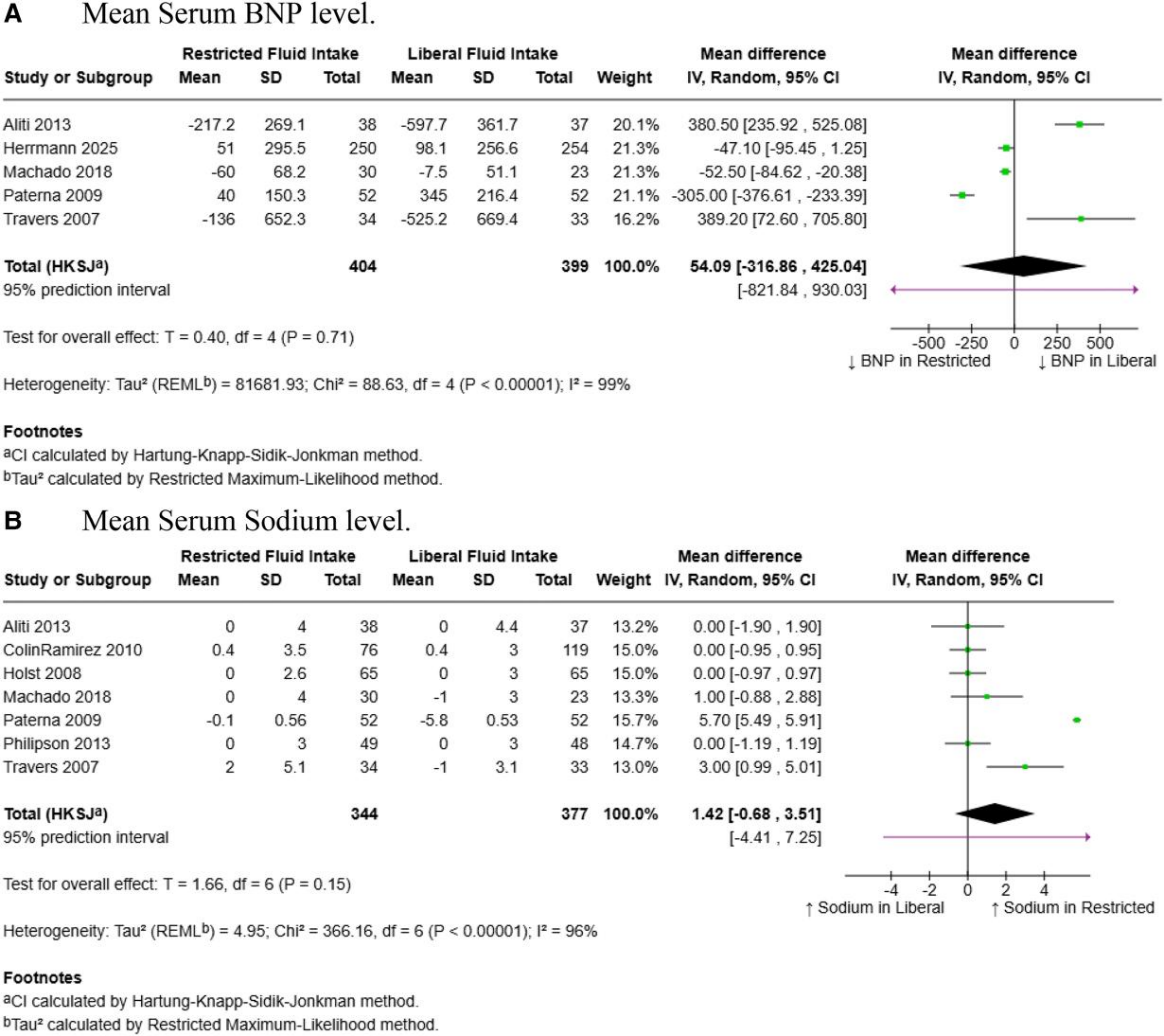
Forest plots summarizing results of (*A*) Mean serum BNP level, and (*B*) Mean serum sodium level

Five out of nine studies were pooled for this outcome. There was no statistically significant difference in serum BNP levels between restricted and liberal fluid intake [WMD = 54.09 pg/mL, (95% CI: −316.86 to 425.04); *P* = 0.71] (*Figure 4*, Panel *A*). The direction of the pooled effect suggested lower BNP levels with liberal fluid intake, but this trend was not statistically significant. Substantial heterogeneity was observed across studies (*I*² = 99%, τau² = 81 681.93), indicating considerable variability in effect estimates. Sensitivity analyses did not meaningfully reduce heterogeneity i.e. *I*² remained >90%.

### Mean serum sodium levels

Seven trials were included in this outcome. There was no significant difference in mean serum sodium levels between liberal and restricted intake groups [WMD = 1.42 mmol/L (95% CI: −0.68 to 3.51); *P* = 0.15] (*Figure 4*, Panel *B*). The pooled effect slightly favored restricted fluid intake, though the result was not statistically significant. There was considerable heterogeneity across studies (*I*² = 96%, τau² = 4.95). Leave-one out sensitivity analysis identified the trial by Paterna *et al*. (2009) as a major source of heterogeneity. After excluding this study, the weighted mean difference in serum sodium remained nonsignificant [WMD: 0.28 mmol/L (95% CI: −0.61 to 1.17), *P* = 0.46], however heterogeneity was eliminated (*I*² = 0%, τau² = 0.00) (Supplementary Fig. S4).

## Discussion

In our meta-analysis based on 9 RCTs showed that there was lower risk of all-cause mortality and no statistically significant lower risk of HF hospital readmissions in FR vs liberal fluid intake. There were no significant differences in BNP levels, sodium levels and thirst between FR vs liberal fluid intake. Dmitrieva *et al*.^23^ assessed the association between chronic hypohydration and HF by analyzing data from Atherosclerosis Risk in Communities (ARIC) study,^23^ population-based prospective cohort study in which 15792 45− to 66-year-old black (African American) and white men and women were enrolled from four US communities in 1987–1989 and followed up for more than 25 years. The study showed that in time-to-event analysis, HF risk was increased by 39% if middle age serum sodium exceeded 143 mmol/L corresponding to 1% body weight water deficit. The authors concluded that chronic subclinical hypohydration could be considered as a new modifiable risk factor for HF.^23^ There are limited studies with small patient numbers that specifically focus on fluid intake in the context of HF.^15–20,24–27^

The pilot study of Holst *et al*.^15^ indicated that a daily fluid intake of <1.5 L was not linked to any discernible benefits compared with liberal intake (30 mL/kg/day) in HF patients postdischarge but water restriction proved to be very challenging to adhere. Overall, none of these have reported a significant effect or association with reduced fluid intake and cardiovascular mortality or HF-related hospitalizations.^15^ Additionally thirst is a very common issue in up to 50% of HF patients significantly impacting quality of life and correlating with prescribed fluid restrictions.^28,29^ Xerostomia, altered taste, dry skin and itching are other side effects of stringent fluid restriction.^15^

In the meta-analysis by Stein *et al*.^30^ fluid restriction significantly increased thirst sensation. In contrast, another meta-analysis conducted by Simão as well as our results did not find significant mean differences in thirst sensation compared with the usual-care group.^31^

In meta-analysis of Vecchis *et al*.^32^ based on 6 randomized controlled trials, patients with restricted fluid intake compared with patients with free consumption of beverages had similar rehospitalization and mortality rates. There were no differences regarding patients’ sense of thirst, duration of intravenous diuretic treatment, serum creatinine levels, and serum sodium levels. Serum BNP levels were significantly higher in the group with free fluid intake.^2^

In a meta-analysis conducted by Stein *et al*.,^30^ 331 HF patients were included from three randomized controlled studies. They demonstrated that fluid restriction alone significantly reduced the relative risk of both all-cause mortality and hospitalization compared with usual care. Li *et al*.^15^ conducted a meta-analysis based on five studies, which demonstrated that fluid restriction has no benefit compared with liberal fluid intake concerning mortality, hospital admission, or thirst in patients with HF.^30^

The inconsistencies in the results of these two meta-analyses may be attributed to methodological differences between the studies. In the recent FRESH-UP study (multi-centre open-label 1:1 randomized clinical trial in chronic heart failure patients on the effect of fluid restriction vs liberal fluid intake on quality of life) among 504 randomized patients (67.3% male) after 3 months there was no significant differences between groups regarding quality of life assessed by the Kansas City Cardiomyopathy Questionnaire Overall Summary Score (KCCQ-OSS).^22^ Thirst distress was higher in the fluid restriction group and no differences were observed for mortality, HF hospitalizations or change in HF pharmacological therapy (for example, initiation or dose increase of loop diuretics) events between the two groups. Our meta-analysis based on 9 randomized controlled trials (including recent FRESH-UP study) showed a statistically significant reduction in the risk of all-cause mortality with restricted fluid intake compared with liberal intake however with no significant difference between restricted and liberal fluid intake groups regarding heart failure hospital readmissions, thirst, serum BNP levels or sodium levels.

### Limitations of the study

One major limitation of the current evidence on fluid restriction in HF is the scarcity of randomized controlled trials for acute and chronic HF, with studies yielding mixed results. The variability in study protocols, heart failure phenotypes, fluid regimen (presented in *Table 1*) and variations in clinical and therapeutic characteristics among studies complicates data comparison and the attainment of definitive conclusions. Patients with severe renal dysfunction were excluded in included studies.

This variability might partly stem from factors such as suboptimal methodological quality, concurrent administration of treatments, small sample sizes, disparate follow-up durations, and climatic differences between regions.

In conclusions, in HF patients, liberal fluid consumption may negatively influence all-cause mortality, but without exerting an unfavorable impact on HF rehospitalizations, thirst, serum BNP levels or sodium levels. Further randomized controlled trials are warranted to definitively confirm the present findings separately for chronic and acute HF. To ensure successful fluid intake, self-care education addressing both the quantity of fluid intake and adjustment of fluid intake based on self-care monitoring is necessary.

For both chronic and acute HF, a more individualized approach regarding fluid intake is suggested. This requirement may vary depending on environmental factors suggesting more liberal intake in hot and humid conditions and/or excessive gastrointestinal losses and fluid restriction in congestion.

## Supplementary Material

xvaf004_Supplementary_Data

## Declarations

### Data Availability

The data underlying this article are available in the article and upon request to the first or senior author.

### Pre-registered Clinical Trial Number

None supplied.
