# Supplementary material for: Fluid restriction vs liberal intake in patients with heart failure: a meta-analysis of randomized trials
Source: ESC Heart Fail. 2026 Jan 8;13(1):xvaf004. doi: 10.1093/eschf/xvaf004 (PMC13108279; doi:10.1093/eschf/xvaf004)
Supplement: xvaf004_Supplementary_Data [file xvaf004_supplementary_data.docx]

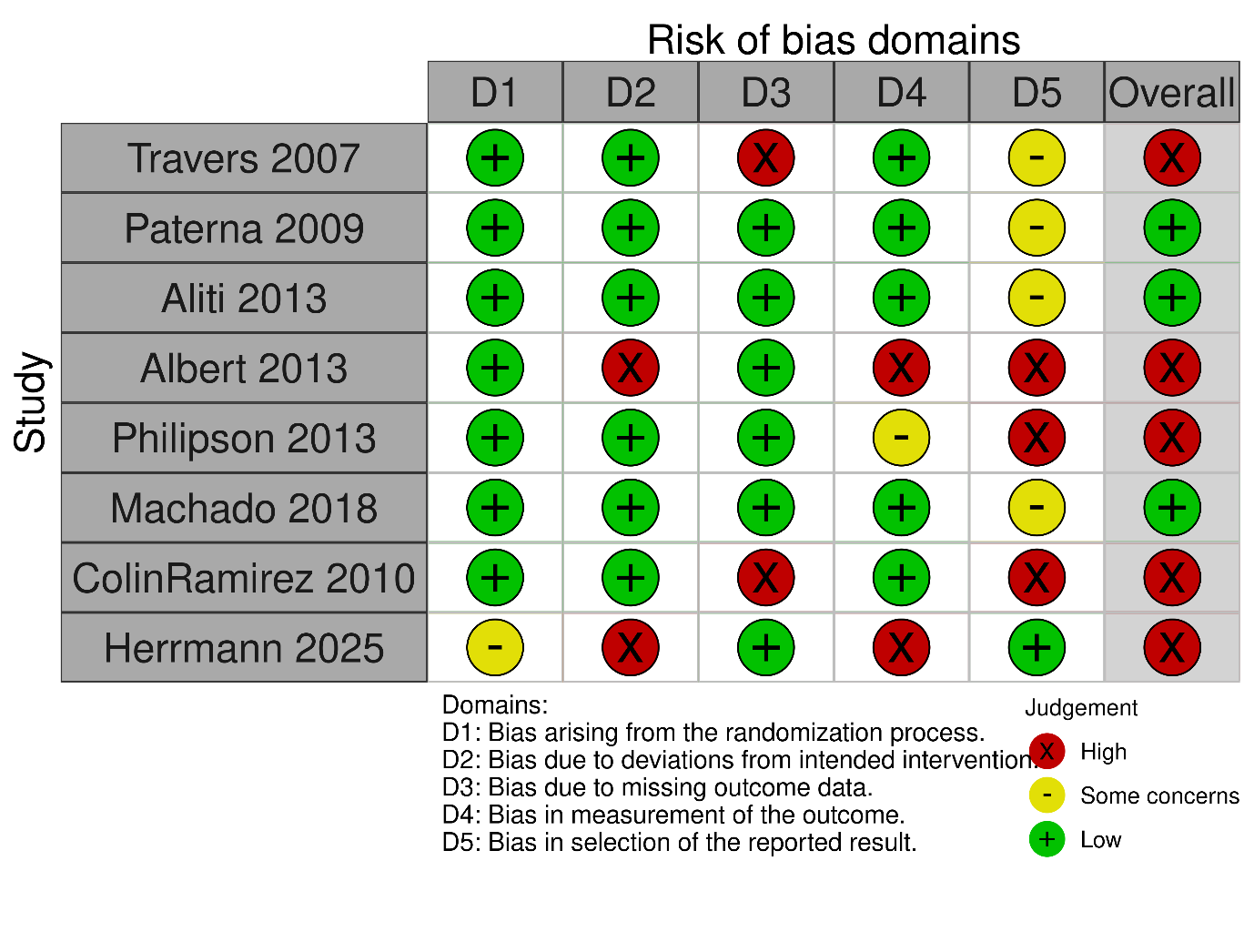


**Supplemental Figure 1:** Traffic Light Plot summarising the risk of bias for randomised controlled trials using Cochrane RoB 2.


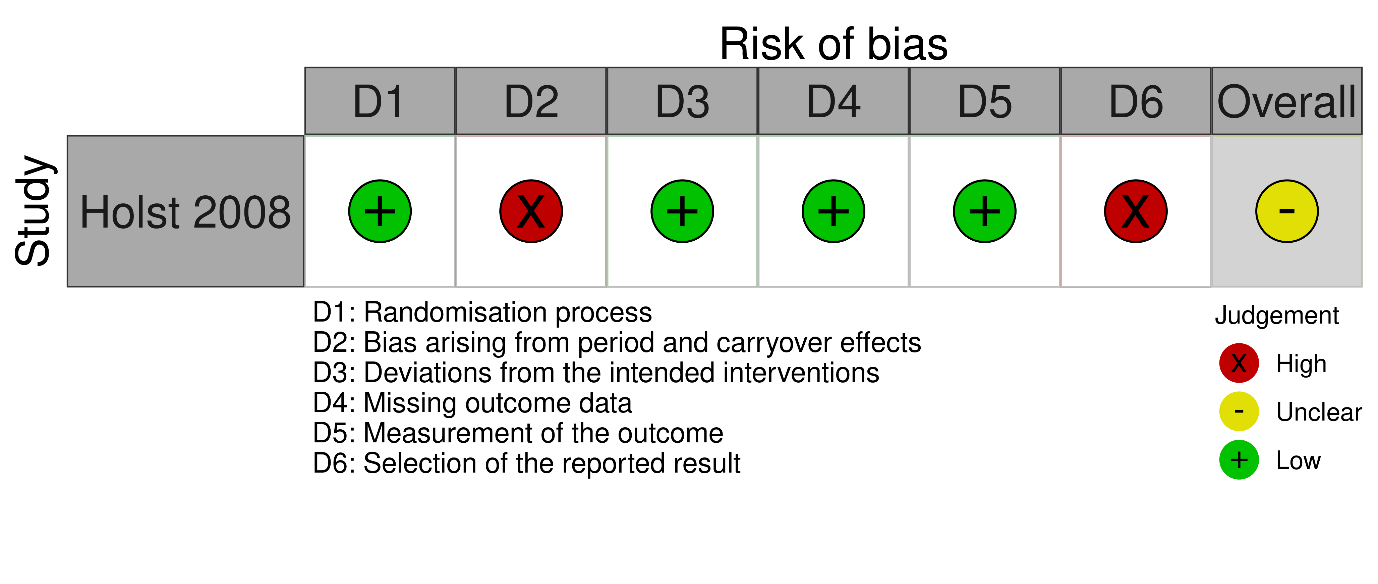


**Supplemental Figure 2:** Traffic Light Plot summarising the risk of bias for randomised cross over trial using Cochrane RoB 2.

**Supplementary Table 1:** Grading of Recommendations Assessment, Development and Evaluation (GRADE) assessment of overall strength of evidence.

| **Authors** | **Agata Bielecka-Dabrowa , Maciej Banach, Danisha Kumar, Vikash Jaiswal** | | | | | | | | | | | |
| --- | --- | --- | --- | --- | --- | --- | --- | --- | --- | --- | --- | --- |
| **Questions** | **What is the impact of the fluid restriction as compared to liberal fluid intake on patients with heart failure?** | | | | | | | | | | | |
| **Setting** | **-** | | | | | | | | | | | |
| **Certainty assessment** | | | | | | | **No of Patients** | | **Effect** | | **Quality** | **Importance** |
| **No of studies** | **Study design** | **Risk of bias** | **Inconsistency** | **Indirectness** | **Imprecision** | **Other considerations** | **FR** | **No FR** | **Relative (95 % CI)** | **Absolute (95 % CI)** |  |  |
| **All cause mortality** | | | | | | | | | | | | |
| **8** | **Randomised trials** | **not serious** | **not serious** | **not serious** | **not serious** | **none** | **580** | **624** | **RR = 0.54; 95% CI: 0.31–0.94** | **-** | **⨁⨁⨁ Moderate** |  |
| **Hospital Readmissions** | | | | | | | | | | | | |
| **6** | **Randomised trials** | **serious** | **not serious** | **not serious** | **not serious** | **none** | **484** | **479** | **RR = 0.67; 95% CI: 0.28–1.65** | **-** | **⨁⨁⨁ Moderate** | **Important** |
| **Perceived thirst** | | | | | | | | | | | | |
| **4** | **Randomised trials** | **non serious** | **not serious** | **not serious** | **not serious** | **none** | **172** | **176** | **WMD = –6.89; 95% CI: –22.86 to 9.08** |  | **⨁⨁⨁ Moderate** |  |
| **Serum BNP levels** | | | | | | | | | | | | |
| **5** | **Randomised trials** | **non serious** | **not serious** | **not serious** | **not serious** | **none** | **404** | **399** | **WMD = 54.09 pg/mL; 95% CI: –316.86 to 425.04** | **-** | **⨁⨁⨁ Moderate** | **Important** |
| **Sodium levels** | | | | | | | | | | | | |
| **7** | **Randomised trials** | **non serious** | **not serious** | **not serious** | **not serious** | **none** | **344** | **377** | **WMD = 1.42 mmol/L; 95% CI: –0.68 to 3.51** | **-** | **⨁⨁⨁ Moderate** | **Important** |

**MD = Mean difference**

**OR = Odd’s ratio**

**CI = Confidence Intervals**

**FR = Fluid restriction**

**
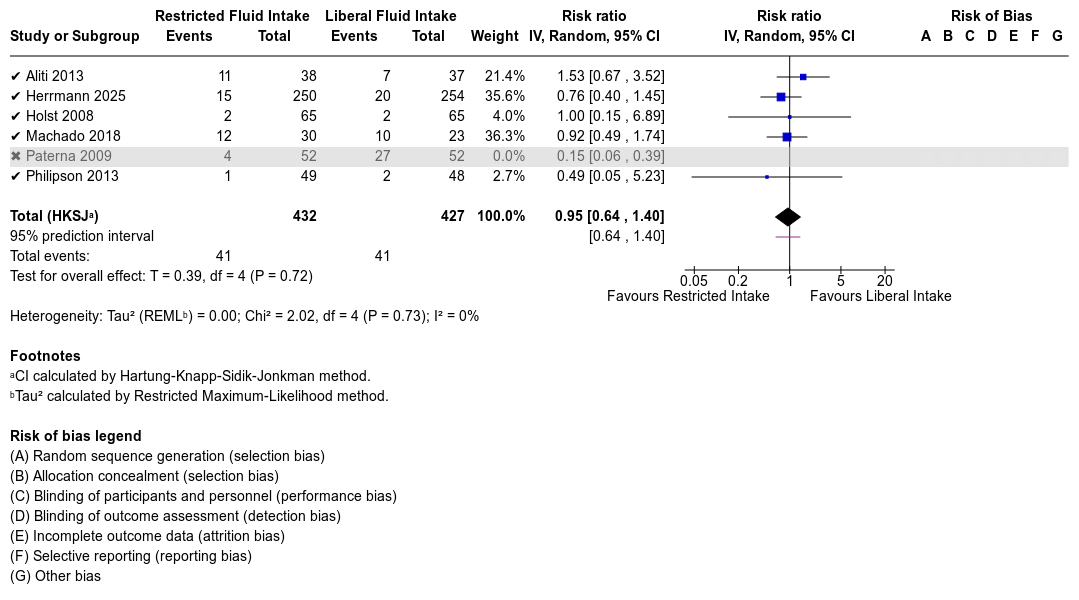
**

**Supplemental Figure 3:** Leave-one-out Sensitivity analysis for hospital readmission outcome

**
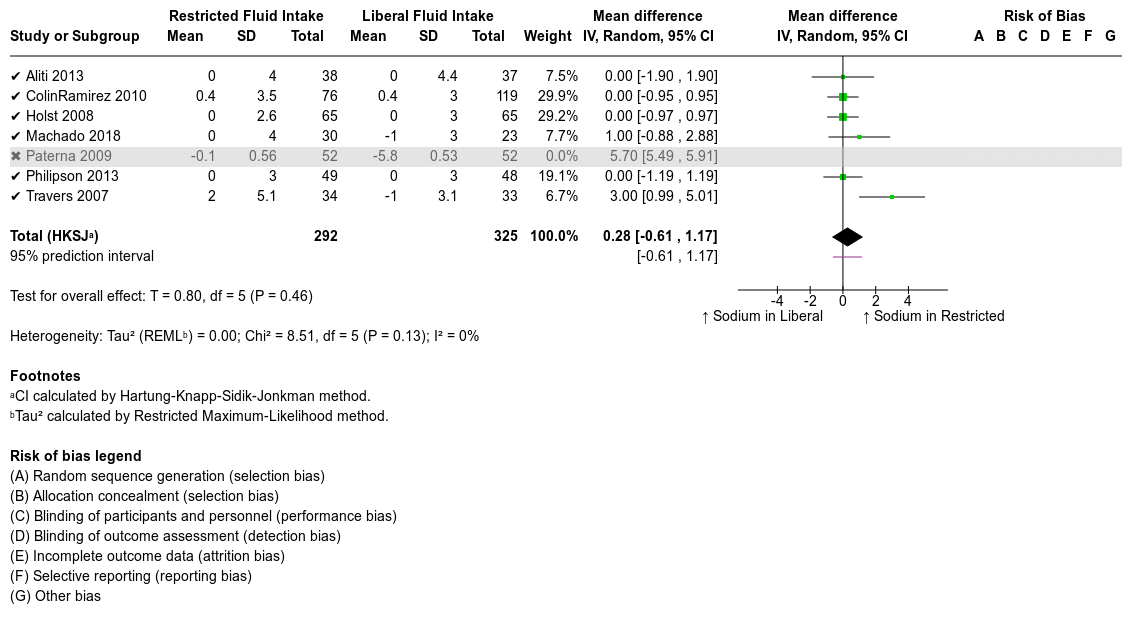
**

**Supplemental Figure 4:** Leave-one-out Sensitivity analysis for mean serum sodium level outcome
